# Retraction: Calvo-Guirado, J.L., et al. The Use of Tooth Particles as a Biomaterial in Post-Extraction Sockets. Experimental Study in Dogs. *Dent. J.* 2018, *6*, 12

**DOI:** 10.3390/dj8030092

**Published:** 2020-08-16

**Authors:** Dentistry Journal Editorial Office

**Affiliations:** MDPI, St. Alban-Anlage 66, 4052 Basel, Switzerland; dentistry@mdpi.com

We have been made aware that Figure 7d of paper [1] is similar to Figure 11a of a paper published in *Applied Sciences* [2]. Both figures show histological images from dogs for experiments carried out in different research projects. The authors were unable to provide an adequate explanation for the similarity. The Editor-in-Chief and the Editorial Office have therefore taken the decision to retract the paper [1].

The Dentistry Journal Editorial Office apologize to the readers of the Dentistry Journal for any inconvenience caused. To ensure the addition of only high-quality scientific works to the field of scholarly communication, this paper [1] is retracted and shall be marked accordingly. MDPI is a member of the Committee on Publication Ethics (COPE) and takes very seriously the responsibility to enforce strict ethical policies and standards.
